# Diaries as Technologies for Sense-making and Self-transformation in Times of Vulnerability

**DOI:** 10.1007/s12124-023-09765-0

**Published:** 2023-04-04

**Authors:** Marcos José Bernal Marcos, Tania Zittoun, Alex Gillespie

**Affiliations:** 1 Faculty of Humanities and Social Sciences, Institute of Psychology and Education, University of Neuchâtel, Espace Tilo-Frey 1, Neuchâtel, 2000 Switzerland; 2 London School of Economics and Political Science, Department of Psychological and Behavioural Science, London, UK

**Keywords:** Diaries, Sense-making, Technologies of the self, Vulnerability, Life course

## Abstract

Diaries have been generally understood as “windows” on sense-making processes when studying life ruptures. In this article, we draw on Michel Foucault’s conceptualization of self-writing as a “technology of the self” and on sociocultural psychology to propose that diaries are not “windows” but technologies that aid in the sense-making. Concretely, we analyzed three non-exhaustive and non-exclusive uses of diary writing in times of vulnerability: (1) imagination of the future and preparation to encounter difficulties; (2) distancing from one’s own experience; and (3) creating personal commitments. Our longitudinal data comprised three public online diaries written over more than twenty years, belonging to three anonymous individuals selected from a database of more than 400 diaries. We analyzed these three diaries by iterating between qualitative and quantitative analysis. We conclude that: (1) beyond their expressive dimension, diaries are technologies that support the sense-making process, but not without difficulties; (2) diaries form a self-generated space for dialogue with oneself in which the diarist also becomes aware of the social nature of her life story; (3) diaries are not only technologies for the Socratic “know thyself” but also technologies to work on oneself, especially in terms of the personal perspective on the past or the future; and (4) the practice of diary writing goes beyond sense-making towards personal development and the desire to transform one’s life trajectory.

## Introduction

People have been writing about themselves for more than two thousand years (Foucault, 1997, 1998; Hadot, 1998). Over the centuries, from Ancient Greece to our current digital era, there has been surprising consistency in the practice of documenting daily activities and thoughts in a diary. Whether in traditional physical notebooks or on web platforms that offer the possibility of developing an online diary (Firth, 1998; Martinviita 2016), many people have a strong inclination to write about their own lives as they live it.

Why do people write down in diaries what they are feeling, thinking, imagining, dreaming, or fearing? What role do diaries play in people’s psychological lives? And more specifically, why do so many people use diaries during moments of transition, disorientation, or reconstruction of their understanding of themselves and the world? In short, how might diaries be technologies for human development in times of vulnerability?

Broadly speaking, one could argue that in both literary criticism and the social sciences, diaries have been generally understood as “windows.” That is, researchers look through these “windows” to access the elements or processes that are of particular interest for the study (e.g., life history, personal beliefs, historical events). However, what if instead of focusing on what the diary allows us to see through the “window,” we turn our attention to the diary itself as a process of writing? Why not focus on the process that diary writing sets in motion, rather than on the outcome that derives from it? Although we are fully in agreement that diaries provide rich data on sense-making dynamics, we think that their value for research on this topic goes deeper. Diaries do not merely reflect sense-making dynamics in motion: the diary is a constituent part of those dynamics.

In this work, we propose a move away from a view of diaries as “windows” on psychological processes in human development toward a view of diaries as technologies to support psychological processes and navigate life ruptures and transitions. For us, these two perspectives are not in opposition. Rather, they constitute different lenses on the same data, suited to answering different questions. In effect, the question shifts from “what do diaries allow us to see as ‘windows’ on psychological processes and sense-making?“ to “how are diaries used for sense-making and what psychological processes are set in motion and amplified by their use as technologies?”.

Therefore, our aim in this article is to analyze the different uses of the diary as a technology for sense-making First, we draw on the theoretical perspective on self-writing developed by Michel Foucault (1997, 1998) in his later years as the basis for our understanding of the diary as a technology for sense-making. Second, we describe the methodology employed in our study, specifying our data: three public online diaries written over more than twenty years and belonging to three anonymous individuals, selected from a database of more than 400 diaries. Third, we analyze three non-exhaustive and non-exclusive uses of the diary: (1) imagination of the future and preparation to encounter difficulties; (2) distancing from one’s own experience; and (3) creation of personal commitments.

### From the “window” to the Foucauldian Technology

Researchers from various disciplines have approached diaries as “windows” on the sense-making dynamics at work under different sociohistorical circumstances: the Spanish Republican exile (Montiel Rayo, 2018), the Nazi extermination camps (Cohler, 2012; Garbarini, 2006), World Wars I (Zittoun, 2020) and II (Gillespie et al., 2008; Zittoun, 2014; Zittoun et al., 2012; Zittoun & Gillespie, 2015), and the Covid-19 pandemic (Hawlina & Zittoun, 2022; Pinheiro et al., 2022). These works highlight the importance of diaries as longitudinal data that give researchers access to the “real-time” development of the person’s mode of dealing with difficult events, to the slow work of making sense of experience until a new form of relative equilibrium is achieved, or to the difficulties of these processes across the life course (Zittoun & Gillespie, 2012, 2021).

In other works, diaries have been approached as “windows” on creativity (John-Steiner, 1997) or specially on issues related to physical and mental health (see, for example, Pennebaker & Smyth 2016; Pennebaker & Stone, 2004). These studies conceive of diaries as an “expressive technique,” focusing primarily on the outcomes of self-writing, that is, its therapeutic healing power and the emotional adjustment (Pennebaker & Seagal, 1999) or maladjustment (Hollis, 2004; Lester, 2004) involved in its use. Without denying the relevance of this emotional expressive dimension, we conceive of diaries as something that goes beyond the expression of thoughts, emotions, and feelings. From our perspective, writing, in general, and diary writing, in particular, can contribute to the qualitative restructuring or transformation of a person’s life experience in a much broader way, articulating it and giving it sense. Consequently, in this article, instead of focusing on what diaries as “windows” allow us to see, we address them as technologies that aid in the sense-making in times of vulnerability.

Our theoretical referent is Michel Foucault, specifically his later works dedicated to “technologies of the self” (Foucault, 1997, 1998). Building on his earlier exploration of the technologies of domination and power, Foucault became increasingly interested in the history of the way in which an individual acts on herself, what he called the technologies of individual domination. In Foucault’s words, these “technologies of the self” permit individuals.

to effect by their own means, or with the help of others a certain number of operations on their own bodies and souls, thoughts, conduct, and way of being, so as to transform themselves in order to attain a certain state of happiness, purity, wisdom, perfection, or immortality. (Foucault, 1997, p. 225)

Foucault understood writing as one of the specific practices used by the ancient Greeks and Romans to improve their own selves. For Foucault, the uses of writing in Greco-Roman culture reflected a decisive change in the thematic orientation of philosophy: the transition from the famous Socratic precept of “know thyself” to another Greek precept, *epimeleisthai sautou*, translated by Foucault as “to *take care of yourself*” (Foucault, 1997, p. 226). Writing began to provoke an inward look, focused on self-care, which amplified personal experience and turned it into something substantive (Blanco, 2002). Foucault explained it this way:

By the Hellenistic age […] taking care of oneself became linked to a constant writing activity. The self is something to write about, a theme or object (subject) of writing activity. That is not a modern trait born of the Reformation or of romanticism; it is one of the most ancient Western traditions. It was well established and deeply rooted when Augustine started writing his *Confessions* […] The new concern with self involved a new experience of self. The new form of the experience of the self is to be seen in the first and second century when introspection becomes more and more detailed. A relation developed between writing and vigilance. Attention was paid to nuances of life, mood, and reading, and the experience of oneself was intensified and widened by virtue of this act of writing. A whole field of experience opened which earlier was absent. (Foucault, 1997, p. 232)

The idea of writing as an act of working on oneself was especially relevant when the person was looking for guidance in moments of crisis. In this regard, the practice of writing provided guidelines for conduct, rules of action, and practical procedures for self-government in moments of personal disorientation (Foucault, 1998; see also Hadot 1998). This working on oneself included, for example, the preparation to encounter future difficulties, a way of imagining and foreseeing hardships and setbacks. According to the *praemeditatio malorum* (Foucault, 1997, pp. 102, 215, 239), when one has meditated or reflected on the possible hard blows of life, and anticipated or imagined them through writing, those blows strike us less hard and wound us less deeply than those that strike unexpectedly. Foucault quotes the following excerpt of the Greek Stoic philosopher Epictetus to illustrate how writing could contribute a disposition of constant vigilance and readiness for anything that may happen in the future:

Let these thoughts be at your command by night and day: write them, read them, talk of them, to yourself and to your neighbour… If some so-called undesirable event should befall you, the first immediate relief to you will be that it was not unexpected. (cited in Foucault 1997, p. 209)

Foucault identified the reactivation over the centuries of a certain number of ancient Stoic writing practices, among them those of the so-called *Hupomnĕmata*. These written documents could be understood in a similar way to today’s notebooks or diaries. They constituted guides to conduct that the individual had to read and reread from time to time to update the teachings contained within. As Foucault explained:

One wrote down quotes in them, extracts from books, examples, and actions that one had witnessed or read about, reflections or reasonings that one had heard or that had come to mind. They constituted a material record of things read, heard, or thought, thus offering them up as a kind of accumulated treasure for subsequent rereading and meditation. (Foucault, 1997, p. 209)

For Foucault, this kind of writing shaped the constitution of the self, understood not as an essence but as a living process that was continuously open to transformation, in permanent change. It was not a matter of extracting a supposed hidden static essence of the self. Writing acted as an echo of everything that the writer had been seeing, hearing, and living in order to establish it as a guide for ongoing conduct:

The point is not to pursue the indescribable, not to reveal the hidden, not to say the nonsaid, but, on the contrary, to collect the already-said, to reassemble that which one could hear or read, and this to an end which is nothing less than the constitution of oneself. (Foucault, 1997, p. 273)

Therefore, following Foucault, writing can be understood as a technique of existence, a mode of subjectivation, a process that requires the person to work on herself to control, test, improve, and transform that self as an open program. Writing is a kind of exercise: “No technique can be acquired without exercise; nor can the art of living, the *tekhne tou biou*, be learned without an *askesis* that must be understood as a training of the self by oneself” (Foucault, 1997, p. 208).

### A Sociocultural Approach to Diaries as Technologies for sense-making and self-transformation

Diarists often write when they experience ruptures that require repositioning and thus create uncertainty about the future. These experiences may even place people in situations of vulnerability, that is, lacking material, social or symbolic resources to achieve a new form of equilibrium or routine (Spini et al., 2017). Drawing on Foucault (1997, 1998), we conceptualize writing as a technology used by the diarists to make sense of their circumstance and to reestablish equilibrium. Following the perspective of sociocultural psychology (Boesch, 1991; Bruner, 1990; Valsiner, 2014; Wertsch, 1991, 1998), with its emphasis on sense-making processes and on the importance of each person’s unique and non-transferable experience, we will conceive of the diary writing as a sociocultural technology for sense-making and self-transformation in times of vulnerability, mediating one’s relationship to oneself and to the environment.

This highlights the dialogical and transitional character of the diary (Zittoun & Gillespie, 2012), which lies halfway between the social world around us and our own way of thinking, feeling, and perceiving. The diary sets in motion a whole work in progress on our inner world as we come in contact with our environment. It is in this transitional space (Winnicott, 1971/2001) between the cultural and the intimate where the diarist elaborates her own experiences, ideas, and feelings in a whole coming and going of voices, perspectives, or scenarios that she can color with her own personal vision or interpretation. As we discuss later in our analysis section, diaries reveal these “others voices within the self” (Gillespie et al., 2008).

Furthermore, we are especially interested in the elaboration of experience that writing may introduce in personal moments of disorientation, crisis, or rupture. We understand that the sense-making process occurs through a regular re-thinking of the past in relation to the present and future, and imagination of alternatives so as to generalize experience and become empowered in the face of a given rupture or crisis (Zittoun, 2020; Zittoun & Gillespie, 2015). Following this idea, diary writing allows people to carry out complex temporal work based on three narrative dynamics characteristic of sense-making processes: (1) linking past, present, and future experience; (2) linking concrete and emotional experience to more general experience; and (3) connecting what is with what could be – in other words, imagination (Zittoun, 2006; Zittoun & Gillespie, 2015).

Considering the temporal narrative work that the practice of diary writing entails, our guiding empirical research question is: If we understand the diary as a technology of the self that contributes to sense-making, what uses of this technology does the person set in motion when writing? Are these uses only involved in the sense-making process or are they also part of a process of self-improvement and self-transformation? In this article, we analyze three uses that, as we specify in the following methodological section, emerged at the intersection of our theoretical framework and the development of our data analysis:

1. *Imagination of the future and preparation to encounter difficulties*: With this use, diarists write about hopes, plans, desires, fears, worries, and doubts. They explore what is, but also what could be, rehearsing the “maybe” or the “what if” of their life stories (Josephs, 1998; Zittoun & Valsiner, 2016). In this way, the work of imagination is oriented toward the future and thus becomes a project or an intention (Pedersen, 2022). Moreover, similar to what Foucault (1997) pointed out, this use might shape the diarist’s anticipated preparation for facing possible future changes or adversities.
2. *Distancing from one’s own experience*: Through the practice of diary writing, the diarist can become aware of certain aspects of her own experience that must be examined or revised (Foucault, 1997). In this *prise de conscience* (Zittoun & Gillespie, 2012), the diarist steps progressively outside of her experience and begins to see her life from new perspectives (Valsiner, 2014; Zittoun & Gillespie, 2012, 2021). In James’s terminology (James, 1890), writing enables the diarist to turn the “I” into a “me,“ taking another step in sense-making development.
3. *Creation of personal commitments*: Writing functions here as a regular (daily or cyclical) ritual of self-reflection, self-examination, or self-analysis of one’s past to change future behavior and orient life in new or better directions. In this case, the diary is very similar to the aforementioned *Hupomnĕmata* analyzed by Foucault (1997): annotations of quotations, reflections, or comments heard or witnessed beforehand, or also elaborated by the diarist afterwards, which serve both as memory aids and as guides to conduct.

## Methodology

We used a mixed method qualitative-quantitative recursive design to analyze the three diaries. Due to the size and multi-year nature of the diaries, we used quantitative methods to “zoom out” and map trajectories and moments of rupture and vulnerability. Then, to better understand the specific uses of the diaries, we “zoomed in” with qualitative analysis, to analyze specific excerpts.

### Database

We had an initial corpus of data drawn from different online diary websites (among others, Open Diary, Prosebox, and LiveJournal). It should be noted that some of these platforms have been active on the Internet since 1995. Despite the great transformations that the Internet has undergone, many diarists have gone on writing their online diaries on a regular basis, as they started doing more than twenty years ago (Martinviita, 2016).

Our qualitative longitudinal data corpus comprised 420 diaries with 11–20 years of writing. We selected twenty-five of these diaries according to three premises: (1) the diary entries were public and did not contain privacy restrictions, such as “member’s only”; (2) they had been regularly active for approximately twenty years; and (3) they were of interest for the study of vulnerability across the life course, containing different ruptures or life transitions.

We first approached this list of twenty-five by identifying their main themes. At the same time, we contacted each diarist through the website’s internal private messaging system. In these first contacts, we gave the diarists detailed information about our research: our names, theoretical perspective, objectives, interests, and procedures to preserve their anonymity. If they were interested, we asked them for their formal consent to use their diary in our research. Finally, once the signed consent forms were collected, we selected the final data sample: three diaries written over a period of twenty years, making a total of 3 million words. The choice of three diaries seemed appropriate given the considerable breadth and complexity of most of the diaries included in our database. These three diaries were downloaded and converted into Excel for analysis.

### Iterative Dialogue Between Qualitative and Quantitative Analysis

Our analysis consisted of an iterative dialogue between qualitative analysis, “zooming in” to provide contextualized insights and particulars, and quantitative analysis, “zooming out” to reveal measures, associations, and statistical patterns. This iterative mixed methods analysis was based on continuous feedback and integration between these two methodological approaches, recursively zooming in and out of the same dataset (Gillespie et al., 2023).

The qualitative analysis consisted of in-depth reading of the diaries using Atlas.ti (Friese, 2019). For each diary, we first elaborated a chronology with the most relevant moments of collective and personal crisis. We also identified sequences of cascading vulnerability over the life course (combination of several events). We began analyzing these three diaries as “windows” on sense-making in times of vulnerability. We used a coding scheme to identify the experiences of rupture and the resources used by the diarist. As we progressed, we had to modify this scheme when we realized that it was impossible to overlook an important fact: the diaries themselves were technologies that these diarists used to try to make sense of their different moments of rupture. Consequently, we modified our analytical gaze to explore how these diaries were being used to navigate moments of vulnerability. This new approach to the data, coupled with our theoretical perspective, revealed the three uses involved in the sense-making dynamics: (1) imagination of the future and preparation to encounter difficulties; (2) distancing from one’s own experience; and (3) creation of personal commitments.

For the quantitative analysis, we used Python, a natural language processing (NLP) program. Using the spaCy tool (Honnibal et al., 2022), this program allowed us to automatically identify people, places, events, or basic themes. We analyzed sentiment in the diary using the VADER tool (Hutto & Gilbert, 2014). VADER (Valence Aware Dictionary and Sentiment Reasoner) is a lexicon and rule-based sentiment analysis tool that is specifically attuned to sentiments expressed in the writing. We used Plotly (Plotly Technologies Inc, 2022), to create interactive plots that could show us at a glance the valence (positive or negative) of the sentiment and its intensity over the twenty years covered by the diary.

Computational analysis of text has become increasingly widespread to measure emotions, cognitions, and beliefs in text (Boyd & Schwartz, 2021; Short et al., 2018). However, it is also imperfect, especially because the meaning of words can change over time (McKenny et al., 2018). Nevertheless, mixing computational analysis with classic qualitative is becoming increasingly common (Guetterman et al., 2018). Specifically, the automated text analysis can guide the qualitative analysis to the most relevant segments, and qualitative analysis can add validity and interpretative depth to the automated text analysis (Gillespie et al., 2023).

**Fig. 1:**
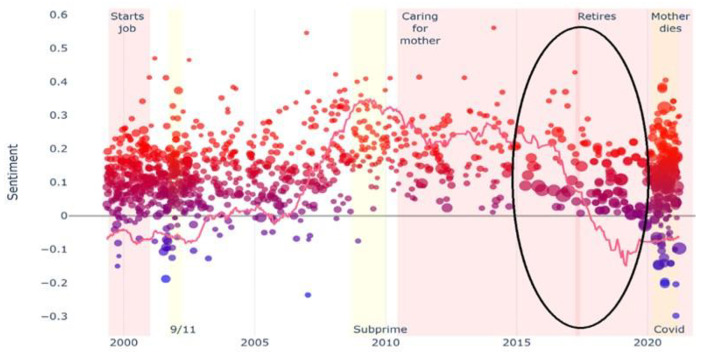
Ernest’s diary visual plot

**Fig. 2:**
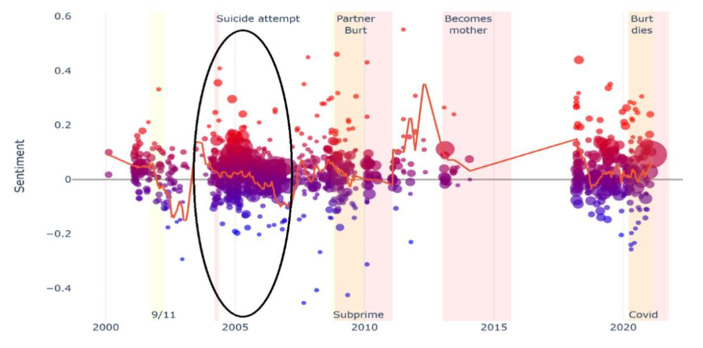
Jeanne’s diary visual plot

**Fig. 3:**
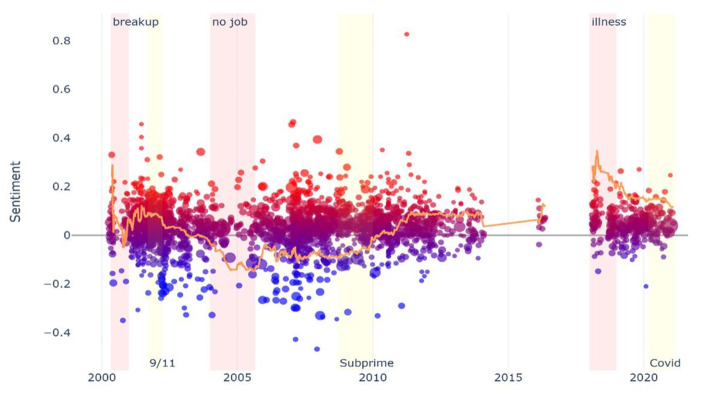
Ken’s diary visual plot

In the visual plots (see Figs. 1 and 2, and 3), the dots located above or below the horizontal axis correspond to entries in which the feeling of happiness (above the axis) or sadness (below the axis) prevailed. The black ovals correspond to the specific moments of vulnerability studied. In Ken’s case, a black oval does not appear because the analysis of his diary did not reveal a specific moment of vulnerability. Instead, we identified a long-term unemployment trajectory of two decades. The dynamics of sentiment detected longitudinally by the NLP program can also be visualized by following the ascending or descending line across the horizontal axis.

### The Three Diaries

We summarize below the concrete events of each diarist that are of interest to our analysis. Each diarist consented to be part of the research and the names are anonymized throughout. As for the entries of each diary that will appear in the analysis, we have decided to delete the dates and show only the year, to preserve anonymity.

Ernest’s diary covers twenty-two years and 1,206 posts. He is a 70-year-old single man with no children. He retired in June 2017, at the age of 65, while he was the main caregiver for his 90-year-old mother, who suffered from diabetes and dementia. His main rupture experience is his retirement (see dark oval in Fig. 1).

Jeanne’s diary covers twenty-one years and 1,041 posts. She is a 39-year-old woman who started writing her diary when she was a 17-year-old student. In this article, we focus on a particular moment in her chronology (see dark oval in Fig. 2): her failed suicide attempt at the age of 21 and her subsequent stay in a psychiatric hospital, where she was hospitalized by her parents against her will.

Ken’s diary covers twenty-one years and 2,282 posts. He is a 44-year-old man who studied to become a filmmaker. In this diary, in contrast to the other two, we did not identify a concrete experience of rupture located in a specific moment (see Fig. 3). We identified a continuous and prolonged experience of rupture, that is, a twenty-year-long unemployment trajectory, with health problems and repeated attempts to give up alcohol.

The three uses are somewhat evident in all three diaries. However, to clarify and synthesize our exposition in line with the length requirements of this article, we draw on one diary to analyze each use. We use Ernest’s diary, when he faces the moment of his retirement, to analyze “imagination of the future and preparation to encounter difficulties.“ We use Jeanne’s diary and her reflection on her failed suicide attempt to analyze “distancing from one’s own experience.“ We use Ken’s diary and his commitment to lead a new way of life throughout his prolonged unemployment, his attempts to quit alcohol, and his health problems to analyze “creation of personal commitments.“

In the following section, we turn to the analysis and how we identified these three uses in the texts. To carry out this operationalization, we rely on an analysis of the content of the diary entries (themes, terms, references to significant others, places, etc.) together with an analysis of textual markers and linguistic cues (verb tenses, changing vocabulary, etc.) (Zittoun & Gillespie, 2021), utilizing the aforementioned back-and-forth between qualitative and quantitative approaches to the data.

Finally, the distinction between the three uses of diaries is primarily analytic. These uses are not mutually exclusive, and they are empirically mixed or connected, as we will demonstrate in our analysis. Moreover, these three uses do not exhaustively or exclusively cover all the possible uses of a diary. Nevertheless, as an exploratory study, we hope that our analysis may give rise to future research that will further refine these uses and perhaps identify additional uses.

## Analysis

### First use: Imagination of the Future and Preparation to Encounter Difficulties

#### Fear and Anxiety Before Retirement

Imagining the future is a common feature in the three diaries analyzed. In Jeanne’s case, for example, her preoccupation with the future has to do with revealing to her parents the reasons for her suicide attempt. In one of the entries written during her stay in the psychiatric hospital after her failed suicide attempt, Jeanne expressed her indecision about sending her parents a letter explaining her suicide attempt in this way: “Should I or shouldn’t I send the letter? […] Jeff said, “you’ll do it when the time is right.“” (Jeanne, 2004). In Ken’s case, the future is associated with his desire to pursue a career in filmmaking or screenwriting. This is demonstrated when he asks himself: “Where do you see yourself in 10 years? Approaching my 35th birthday. Hopefully making films or writing” (Ken, 2003). In both cases the diarists are future-oriented, deciding when to act and imagining possible futures.

To examine this future orientation in more detail we will focus specifically on the case of Ernest, whose vision of the future is mainly associated with his constant reflection and concern about retirement. We identified imagination of the future and preparation to encounter difficulties by detecting entries in Ernest’s diary that had to meet the following requirements: (1) they were written prior (years, months, or days) to his official retirement, which occurred in June 2017; (2) they were entirely devoted to reflecting on the specific future event of his retirement.

Following these concrete criteria, analysis of the content and linguistic cues associated with his reflection on retirement revealed two significant points. First, retirement begins to be a frequent theme in Ernest’s diary up to twelve years before his official retirement. For example, in a 2005 entry, he asks himself: “Where do you see yourself in the future, say – 10 years from now? I see myself doing my present job and nearing retirement and being totally and ecstatically ready for it, but other than that I have no idea” (Ernest, 2005).

Second, the mention of retirement in his diary is frequently associated with a reflection on mixed feelings. On the one hand, Ernest writes about his desire to free himself from the obligations and routines associated with work. On the other hand, he also mentions his doubts, insecurities, fears, and anxiety about losing his main source of social life, especially being single with no children and caring for his 90-year-old mother, who suffered from diabetes and dementia.

As retirement approaches, Ernest’s entries reflecting on his future retirement are progressively frequent. The trend remains the same as above, marked by desire, idealization, doubts, and insecurities. Take, for example, this excerpt written in 2014, three years before his retirement. Note how he perceives his doubts and the coming change, while he romanticizes the retirement describing it as leisure time to sit down on the porch and rest:

Last week I was saying I could work, and, might HAVE to work indefinitely until 70 or more. This week I want so badly the leisure time and peace that comes with being able to sit on the porch all morning with my coffee and not have to scramble to get to work. And work seems to be less necessary for me now to be the person I have become and meant to be. (Ernest, 2014, diarist’s emphasis)

#### Imagining a Purpose: Facing Retirement as a new Beginning

While idealizing his retirement, Ernest uses his writing to prepare for that future event. This reminds us of Foucault’s comments on the Stoics’ writings as a preparation exercise for encountering probable future difficulties. In a similar sense, Ernest uses his diary to refuse to internalize the cultural narrative of decline frequently associated with retirement (Freeman, 2011; Bernal Marcos et al., 2022). Through his writing, he is already projecting a way of feeling toward this vital moment: to understand it not as an end, but as a beginning. This is how he describes it below:

And now, life goes on, the job is not as fresh, and I see retirement looming ahead. When will it be? Where will I go to live? What will I do? That’s what’s exciting about it. All those big questions to answer and a time in which I will start over near the end of life. Endings into beginnings. (Ernest, 2014)

In this quotation, it is interesting to note how two important points are combined linguistically. On the one hand, Ernest poses three rhetorical questions, in which he imagines his future retirement as something uncertain and unknown. On the other hand, his use of the future tense indicates the establishment of a purpose: “I will start over.” This is a textual marker that implies a kind of commitment to oneself and anticipates the third use that we address later.

Three years later, only a month and a half before his actual retirement, Ernest’s writing continues to deepen his commitment to facing retirement as a new beginning and not as a “narrative foreclosure” (Freeman, 2000) – as an opportunity to reinvigorate his life story and continue to grow, learn, and develop as a person. The following excerpt precisely reveals this desire or conviction:

I have no idea what will happen in the next year. I can make plans but who knows what will come of those? I’m suddenly pondering the mysteries of life in ways I never have before. I know one thing: retirement will free me to engage in my lifelong quest for knowledge, a deepened faith, and the exciting possibilities of unlocking doors to the unknown, the mysteries of the universe. I truly believe that and look forward to that time, even though it still seems unreal that I am even contemplating all this. (Ernest, 2017)

#### Diary Writing as an Awareness on the Social Dimension of our Lives

Up to this point, we have seen how the diary forms a space in which Ernest works out or tests his thoughts or feelings about his future retirement and possible actions. As he writes, Ernest explores or imagines future life scenarios, establishing a purpose, an attitude, and a way of feeling toward retirement. However, the space created by the writing also includes an awareness of the social dimension of the self and the plurality of voices that shape one’s life story. Accordingly, the exploration of the future that takes place in Ernest’s diary also includes a dialogical and social dimension. In this sense, Ernest is anxious about his retirement because, as he confesses, he will lose his main source of social contact:

When I think of no longer working, I get this very strange and anxious fear of the unknown. Even now, beforehand, I feel my life has suddenly been shortened, that I am older than I would, or could, ever really acknowledge until now, and that I am going to embark on the final stage of life alone, with no immediate family[…] I’ve made a lot of good friends in past jobs, but I’ve basically been a solitary person all my life, and this job is now my only social outlet. Leaving it will create a tremendous vacuum. (Ernest, 2017)

Ernest writes particularly about those “others” who not only participate in his life story, but also shape it. This quote is also an example of how Ernest reflects on his own life and defines himself as “a solitary person,“ aware of the “tremendous vacuum” that retirement may entail in his life course. This *prise de conscience* (Zittoun & Gillespie, 2012) reveals, on the one hand, Ernest’s intention to face retirement in a certain way and, on the other hand, his doubts, vertigo, and fear about it, as he describes below:

After 22 years of coming to the same workplace and the same comfortable, book-filled and paper strewn cubicle, I am retiring (or at least think I am. I have two days to change my mind). It’s hard to even say it, much less write these words[...] This is big. This is serious. In a short while, my life will be dramatically different, or at least I think it will be now as I write this. (Ernest, 2017)

In the process of imagining the future, the writing moves continuously between looking to the future (the diarist wonders what is going to happen, how he is going to feel, how he should face the future) and looking to the past (the diarist feels vertigo, becomes aware of the change or rupture that a future event may imply in his life trajectory up to the moment he is writing). In this oscillation, Ernest’s writing successively sets in motion an idealization of retirement, a purpose, and an awareness full of hopes, doubts, and fears about the rupture that such an event might entail. Through this awareness, his writing also contributes to a distancing from his own experience, an aspect that connects this first use with the next one we address below.

### Second use: Distancing from One’s own Experience

#### A Dialogue Prolonged in time: The Diary as a Rendezvous with Oneself

In the three diaries analyzed, the exercise of writing makes the diarists articulate certain aspects of their experiences that need to be examined or cared for. Their diaries can be understood as a *rendezvous* with themselves in which they reflect repeatedly on issues of special relevance in their lives, distancing from their own experiences. Ernest, for example, continually reflects on his retirement. In his diary we can identify a progressive change in the consideration of this vital moment. It is anticipated with anxiety and fear, then described as a crisis that leads to depression, and finally reconceptualized as follows: “It’s been 2 1/2 years since I retired, and that momentous milestone was one of the best things that ever happened to me” (Ernest, 2020).

In Ken’s case, there is repeated reflection on his situation of unemployment and the frustration he feels at not being able to pursue a career in filmmaking. Ken writes about his failed attempts to find a stable job in the film industry, leading him to reread his past entries to take perspective on his own experience. In an entry entitled “Looking back” he writes:

Do you want to know what I’ve just been doing? Re-reading old entries in this diary […] I am going to just look back once every so often […] I know that you are not supposed to dwell on the past. But I think that it could be good. It may even be cathartic. It’s reasured me that the way I’m feeling right now is not new. I have felt like this before. (Ken, 2001)

In this section, we focus on the diary of Jeanne, a 39-year-old woman who attempted suicide when she was 21 years old and was subsequently hospitalized by her parents in a psychiatric hospital. We identified the distancing in the diary by analyzing: (1) the entries written by Jeanne while she was hospitalized; and (2) the entries written on the anniversary or dates close to the anniversary of her suicide attempt. In this regard, we should clarify that, amidst the diversity of content in Jeanne’s diary, we identified a kind of annual *rendezvous* with herself. On the anniversary of the failed suicide attempt or around that date, Jeanne writes a reflection on both the attempt and her experience in the psychiatric hospital. This is how she explains this continuous dialogue with herself over time and the need to write and reflect on those experiences:

Always happens in the spring, as we near the anniversary[...]and I start getting more lunar. So yes, in April, I always start thinking about suicides. Not necessarily my own[...]but just what it means to be alive by accident. I used to call every special occasion for the following year after my attempt “The Bonus Round”. It was the year I wasn’t supposed to have. (Jeanne, 2009)

Jeanne’s writing clearly reveals a need to return again and again to those decisive life events, a continuous need to shed light on those moments to make sense of what happened to her. We now look at how Jeanne’s progressive construction of a rich and more nuanced narrative takes place.

#### Struggle for sense-making Through Writing

Jeanne’s writing about the decisive life events mentioned above changes over time, revealing a progressive distancing and new perspectives. During her hospitalization and in the years immediately following, we identified a writing that we describe as “a vicious circle,” with Jeanne trapped in a constant search for sense that is very difficult and fruitless. Progressively, Jeanne begins to elaborate through her writing a more nuanced, broad, and distanced look at her own experience. This includes the reconceptualization of her suicide attempt as a key resource for interpreting her life story.

To analyze this aspect, we selected one of the excerpts written by Jeanne in the psychiatric ward in May 2004 and diary entries written in 2008, 2009, 2018, and 2022 on the anniversary of her suicide attempt. This selection was based on the relevance of these excerpts to the slow and laborious sense-making that Jeanne is engaging in through her writing. We identified significant variations across these entries in terms of both content and form, i.e., thematic or linguistic markers revealing the process of distancing: topics, vocabulary, descriptions, metaphors, verb tenses, etc.

According to Jeanne’s description, during her stay in the psychiatric hospital, she was not allowed to carry paper or a pen. Even so, she managed to hide a notebook and write in it, transcribing the complete notebook entries into her online diary after her release from the hospital. We can get an idea of her state at that time from the following lines written right at the moment:

Things aren’t all bad here. They’re beyond bad. Putting a suicidal person in a mental hospital is like putting raw sewage thru a blender-it doesn’t make sense, it doesn’t work and it stinks. I mean, just the fact that I’m young and impressionable when I’m suicidal, makes me vulnerable. The crazies kinda bring me down to their level. (Jeanne, 2004)

In this excerpt Jeanne’s use of the raw sewage metaphor underlines the fact that the situation does not make any sense to her at all. She seems completely disoriented. Through writing, she tries to convince herself that she is not like the other patients, whom she calls “the crazies.“ She seems to wonder: Do I belong in this place? What am I doing here? Am I on the same level as them?

In the years following her suicide attempt and her stay in the psychiatric hospital, Jeanne tries to rebuild herself after hitting rock bottom. She uses the diary as a way of asking herself what had happened to bring her to the point of attempting suicide and ending up being locked up by her parents in a psychiatric ward. Our analysis detected a specific theme around which the sense-making dynamics revolve: the realization that she was miraculously alive. We must specify here that, according to Jeanne’s description of those events, she came out of the attempt alive because one of her roommates had accidentally discovered her lying on the floor and had managed to call an ambulance to save her life. On the fourth anniversary of that date, Jeanne writes: “Usually I sit there on my anniversary of my suicide attempt and wonder why I am still here when I quite clearly was not meant to be. Then I sit and feel bad about myself. Ungrateful” (Jeanne, 2008).

A year after the previous excerpt, the event still makes no sense to her. Jeanne is still trying to make sense of the fact of being miraculously alive. This can be clearly seen in the following excerpt written on the fifth anniversary, in which she describes herself as a person who is still limping:

5 years ago on this day I almost ceased to be. I can’t believe it’s been so long[…] I am still waiting for that break to heal but I just keep limping. 5 years later I don’t know what’s changed. Time doesn’t make sense anymore. Nor does my life. Though I know I have talked about this memory a lot it still isn’t enough b/c it doesn’t make sense. It has to make sense. I have become obsessed with making it work. (Jeanne, 2009)

Note the use of the adverb “still” on two occasions, associated with waiting “for that break to heal” and waiting to find sense – waiting that becomes an imperative (“It has to make sense”) and even an obsession (“I have become obsessed with making it work”). In a Foucauldian sense, Jeanne continues to work on those events through writing, thinking about them, questioning them, meditating on them obsessively (in Jeanne’s own words). However, Jeanne’s perspective remains that of someone who is striving to make something clear, but who continues to see it as a blur.

However, in our analysis of the subsequent years, the slow narrative elaboration of that experience begins to change. Jeanne’s writing seems to extract some sense, little by little, very precariously, and very slowly. Let us take an entry written on the fourteenth anniversary as a representative example of this new trend. A new element of content appears in Jeanne’s writing as, for the first time, she describes her suicide attempt as a lesson of life:

Today I am feeling reflective. Fourteen years ago today, I nearly didn’t make it to see May 5. […] In recent years I have become more open about my suicide attempt, because I feel there’s a lesson worth learning here & sharing our life experiences is the currency of knowledge. I think about how if I had died, I wouldn’t have gotten to grow as a person. I would’ve forever been remembered as that idiot kid […] who said and did things she would never say or do now […] I think about all the books I have read, all the movies I’ve seen, all the music I’ve fallen in love with in 14 years. That is a lot of material to experience, to absorb, to love […] On the worst days, if I think of where I could be, if not for the mad luck of a friend coming to check on me and saving my life, I have to admit that I like the view here much better. (Jeanne, 2018)

The complete entry is an elaborated reconsideration, a new narrative to make sense of her failed suicide attempt and her whole life. Note the predominant use of the subjunctive mood instead of the indicative. Her writing functions here as a glimpse into her adolescent self, that “idiot kid” who she now believes would behave differently. But Jeanne also imagines through her writing all that would have been lost in her life had she been dead. There is an emotional development shaped through her writing that we can identify in the tone of the writing and the mood and feelings she mentions. In the same entry, she writes: “There are days where it is a struggle to be my best self […] I just try to remember that there is still so much love to refract in and out through this human body” (Jeanne, 2018). Now, her use of the adverb “still” no longer refers, as we saw before, to a desperation to heal, but to a feeling of hope for new future horizons.

#### Finding Sense and Purpose in the Others

On the eighteenth anniversary, Jeanne’s reconsideration reappears. The psychiatric hospital, which she described in previous diary entries as a senseless place, is now defined as “the place where I first got an idea about how to help myself &, ironically, it was not in any therapy session” (Jeanne, 2022). The interesting point here is to note how this reconsideration is now linked to the social dimension of that hospitalization. Again, as we saw earlier with Ernest, Jeanne’s writing reveals an awareness of the role of others in her life story. In a long entry full of anecdotes, she recalls her companions in the psychiatric ward: “I wonder if they’re ok. I wonder if they beat the statistics of failed suicide attempts like I did or if they finally got their wish to move beyond this earth” (Jeanne, 2022). This new reconceptualization is prompted by the memory of the first encounter with one of her companions, a lady with whom she played Scrabble. In that memory reconstruction, her writing identifies what she calls “a glimpse of something life-changing for me”:

There was a lady there[...] She seemed confused and completely removed from reality and I watched her, terrified, every day because at that point, I didn’t know if I would become so crazy that I would become like her. The staff made me play Scrabble with her once and she just made up words. Tamoot. Yglib. I didn’t challenge her, I just let her play them-it didn’t matter. And just sitting with someone that I could still show kindness to and help, even in my advanced state of brokenness, was a glimpse of something life-changing for me. It was amazing to realize that I wasn’t too far gone, too mental. I could still do good, contribute in ways that were meaningful. I could take care of others, even if I couldn’t take care of myself. (Jeanne, 2022)

Note the contrast of this excerpt with the one mentioned above, in which Jeanne describes the psychiatric hospital as a senseless place and refers to her companions as “the crazies.“ Now, her view of those “crazies” is very different. She sees what she shares with them and recognizes the following: “I mean, let me be straight, I think I’m just as crazy as the rest of them[…]I’ve just been able to find ways to continue to function” (Jeanne, 2022).

Finally, it is interesting to note how this broad reconsideration not only manages to construct a new sense around that experience and her companions, but also gives sense to her subsequent professional dedication as a nurse, as she notes in the following excerpt:

I realized that the best possible thing I could do for myself to heal was to take care of others[…] that the way I could function and find purpose, was to take care of others who needed help. It’s no surprise that I have worked at a nursing home with people with dementia & now with people with disabilities for all of my adult life. It has been a hard road and there will always be a lot of sadness & rage & suicidal ideation & mania-because that’s my brain, because both nature & nurture were rabid, diseased cunts to me. And that’s ok. Because I honestly believe taking care of others has been the only thing that has helped me to function and remain upright in spite of all my other fuckedupness. Sometimes when I think about the hospital, I wonder if the other ladies who were there with me figured that out, too. I hope so. And I hope that they have reached the Someday where someone asks them if they are happy to have survived & they answer yes. Immediately. Without waiting. I know I have. Go ahead. Ask me. Yes. (Jeanne,2022)

In conclusion, in Jeanne’s diary, we see an evolution of distancing through time as she holds an annual *rendezvous* with herself on the anniversary of what could have been her death by suicide. At first, Jeanne painfully notes her frustration at failing to shed light on those events. She says she has become obsessed with trying to make sense of the fact that she is miraculously alive, but she cannot. Later, her writing transforms the valence of those events that have shaped her life, contributing to the configuration of a new sense. That difficult experience is then reconceptualized as: (1) a key resource for interpreting her own life story; (2) a key narrative element to make sense of her later professional dedication as a nurse; and (3) a resource for reconsidering the importance of the others.

### Third use: Creation of Personal Commitments

#### Diary Writing as a self-examination

Moments of self-examination that lead to the establishment of personal commitments are common in the three diaries analyzed. In Ernest’s diary, for example, his regular walks through the countryside lead him to formulate his own commitment to a simple lifestyle connected to nature:

I’m not going to hope for too much. I am going to be content with the everyday pleasures that make life satisfying and more complete […] I marvel every time at the grand old live oaks. I watch the egrets soar over the small lake when I sit on a bench overlooking the water. I see dark clouds part and sunshine coming through. These are joy enough for me. (Ernest, 2008)

In Jeanne’s case, her commitments are formulated with her usual dose of irony and sarcasm:

What a Beautiful Rock Solid Foundation. Let’s Review:

New Year’s Resolution 2004: QUIT LIFE.

New Year’s Resolution 2005: QUIT DRINKING & SMOKING.

New Year’s Resolution 2006: QUIT EATING.

New Year’s Resolution 2007: QUIT PUKING AFTER EATING.

New Year’s Resolution 2008: QUIT QUITTING.

New Year’s Resolution 2009: QUIT FAMILY. (Jeanne, 2009)

In this section we will focus specifically on Ken, who studied to become a filmmaker and experienced a twenty-year-long unemployment trajectory. We identified personal commitment by detecting specific moments of self-examination in the diary. These moments are accompanied by textual markers that establish this diarist’s intention to amend his own way of being or acting. Generally, these textual markers correspond to expressions such as “I am going to,” “I will,” or “I must,” which mark the desire to start over and a goal of personal evolution and transformation.

In Ken’s case, such expressions are related to a process of personal disorientation amidst a prolonged unsuccessful job search that led him to define himself as a long-term unemployed. According to his own diary entries, Ken feels lost, constantly looking for a job and spending the day drinking more than he thinks he should. He very sporadically finds temporary jobs, but these are never stable or related to filmmaking. Ken goes through periods of frustration owing to his fruitless job search. Through his diary writing, he is continuously rethinking his own circumstances and his own path in life (Zittoun et al., 2022). He reflects in the diary on different aspects of the job search process and sometimes, as in the following excerpt, he writes down these commitments:

“I feel like I’ve been in a coma for the past twenty years. And I’m just now waking up.” Lester Burnham, American Beauty.

As you may have noticed, I have changed my diary. Old entries will also be changed so that every entry (except jokes) will start with a quote. Why am I doing this? It is the first step to me changing myself. I am going to start working out and cut down on the amount of alcohol I drink. I WILL finally get a tatoo and I am even thinking of dying my hair Eminem white.

Through this a positive plan I aim to start projecting attitude about myself. If I do this I may even start thinking positive things about myself. I will get my script finished and someone will love it enough to green light it and I will then be involved in the making of films which is what I wanna do. I am not a victim. (Ken, 2000)

With respect to this commitment, it is interesting to note, first, how the entire entry is preceded by a quotation from the film *American Beauty*, just as the ancient Greek *Hupomnĕmata*, mentioned by Foucault (1997), included quotations or excerpts from books read or conversations heard as personal guides to conduct or teachings. In Ken’s writing, the words of the film’s protagonist summarize or describe his own life and his own desire to awaken and transform himself (“the first step to me changing myself”).

Second, note how this look into the future is not the same as the one analyzed in our first use. Ken’s writing does not focus on a specific future event that he imagines or for which he claims to be preparing, as was the case in Ernest’s writing, which was full of doubts and fears in preparation for his retirement. The new look into the future elaborated in Ken’s diary stems from an examination of his past conduct, which gives rise to a commitment to himself to do things or conduct himself differently in the future, to improve his life, or to even transform it. Note how he writes “I am going to,” “I will,” and “I aim to.” In turn, this self-acquired commitment acts as a kind of continuous self-monitoring of one’s own will, one’s own path, or one’s own performance or orientation in life.

Third, this use underlines the active character driven by the diary writing. Ken’s written resolution commits him to a new way of acting. He wants to change himself, to transform himself, to become someone else, to take control of his own development or becoming. Through writing, Ken comes to understand himself as someone who, up to that moment, has behaved as a victim. However, in Ken’s resolution, he no longer wants to be at the mercy of circumstances. He wants to take responsibility for his own life, to face it differently, and to feel and think about it differently. He writes with conviction: “I am not a victim.”

It is also interesting to note how the intended personal change begins with a change in the appearance of his diary, as if the very instrument through which he formulates his new resolution should also change to bring about the change in himself.

#### A Commitment in the Presence of the Other

Another interesting aspect with respect to Ken’s commitment is the social dimension. It is interesting to note how he addresses his regular readers, with whom he interacts frequently. As Foucault notes when referring to the epistolary writings of the Stoics (see, for example, Foucault 1997, pp. 214–221), the exercise of writing for another is also a form of exercising, monitoring, and examining oneself. A year after the above excerpt, Ken is still trying to stop drinking, and his resolution to make amends is again a shared commitment and a shared dialogue, in which he asks the reader to wish him well:

I have decided to give up drinking for a week...The main reason is that I am tired of waking up in the morning wondering what the hell I did the night before. Feeling rough. So I’ll try it for a week. If it works out, I will stay teetotal. Well, with the exception of wine with meals. And new year. And my birthday. Wish me luck. (Ken, 2001)

Years later, in addition to problems around unemployment and drinking, Ken begins to talk about health issues. Again, it is in the diary that Ken proposes a change to his life. The commitment to a new way of life involving swimming is again formulated in the presence of others in the following way:

So, I’ve decided it’s time to get shot of the Buddha belly. Can’t really call it a beer belly any more, really. With my particular health issues, I decided that swimming would be the best idea... Go swimming for an hour, that’ll be easy. Except when I went swimming as a teenager, it was more like just messing around in the pool. I probably swam for a grand total of 10 minutes then had to give up. Going to keep going with it, though. (Ken, 2019)

Once again, there are linguistic markings that reveal how Ken commits himself to do certain things and to take care of his health: “I’ve decided,” “Going to keep going.” The following year, the commitment is still active. Ken’s writing reflects on the benefits of swimming for his personal functioning and for dealing with life, including his work and health problems:

I’m really enjoying my swimming. I’m not going to say it’s relaxing, that’s not the point of me being there. But, I’m usually tired when I leave and that is why I’m there. I’ve noticed that my stamina is increasing as well... It’s also good for clearing my head. (Ken, 2020)

In sum, Ken’s shared meditations or soliloquies constitute, like the ancient *Hupomnĕmata*, guides for conduct, rules of action, and practical procedures for self-government in moments of crisis or disorientation. In his diary writing, Ken continuously takes up the always unfinished, always provisional task of exhorting, examining, persuading, and criticizing himself. In short, as Foucault (1997) supposed when analyzing the Stoics, self-writing contributes to the attempt to become aware or to remain as awake and as lucid as possible with respect to the always unfinished and problematic task of living – in an attempt to try to make sense of what is happening to us, to try to change ourselves, our own way of thinking and feeling, and our own life perspectives.

## Conclusions and Openings

### The Diary Beyond Emotional Expressiveness: Sense-making Dynamics and Possible Difficulties

Diary writing encompasses functions that go beyond the expressive dimension. A diary is not only an emotional receptacle where thoughts, feelings, fears or hopes, previously elaborated by the diarist when experiencing situations of vulnerability, are clearly and neatly expressed. The analysis shows us that it is precisely the activity of writing itself that helps the diarist to make sense of the experience of vulnerability, clarifying it, elaborating it, rethinking it in one way or another as she writes and reflects on it. The diary writing itself contributes to understand one’s own ongoing experience as it is lived.

Therefore, in difficult moments of crisis, it may be necessary to consider the role of diary writing more carefully as a support or tool of the sense-making processes. It may also be necessary to study, as a future research direction, those moments in which writing does not seem to work, that is, its limitations in making sense of life ruptures or traumatic experiences. In Jeanne’s case, for example, we see precisely how writing places her in a vicious circle from which she finally emerges, not without difficulty, many years later. Following Pennebaker’s warnings (Pennebaker & Smyth, 2016), it is necessary to pay attention not only to the potential positive effects of writing, but also to its possible detrimental effects on certain people or on different circumstances.

### The Creation of a Dialogical Space: Diary Writing as Social Contact with Oneself

In the three cases analyzed, the practice of diary writing carves out a self-generated space for dialogue with oneself. Ernest, Jeanne, and Ken have created a written conversation with themselves that has lasted for more than twenty years, in which they question their life trajectories in dealing with personal moments of vulnerability. This dialogue with oneself is, at the same time, a dialogue with the other voices that populate their life stories – a personal and cultural, intimate, and collective crossroads. In the three uses, the diarists become aware of the dialogical and social dimensions of their own lives. In this sense, we could say that self-writing is a form of social contact with oneself.

### Foucault’s Trail: Diary Writing as a work on Oneself in Times of Vulnerability

We have seen that the practice of diary writing is not only a Socratic know thyself”. As Foucault suggested, the appointment with self-writing implies care of and work on oneself. In the three diaries analyzed, the diarists’ writing is an impulse to look at their own personal trajectories, setting in motion a complex process of self-examination, self-care, and self-awareness – a process that is always provisional and transitory, in a continuous dynamic of making and renewing itself. We have seen how the diarists examine their actions, thoughts, or feelings – working on them, clarifying, or elaborating them through the process of writing in their diary. In times of vulnerability, this practice helps them to see more clearly or gain awareness of certain aspects of their lives.

In Jeanne’s case, for example, she works on her perspective on the past, even creating a space in her diary for an annual reflection on a decisive crisis to which she returns again and again. Through the exercise of writing, she distances herself from her own experience and begins to develop a new perspective on those events. In Ernest’s case, the writing works on his perspective on the future, preparing him in anticipation of his both feared and desired retirement.

### ”You must Change your life”: The Diary as a Vygotskian zone of Proximal Development

The practice of diary writing can go beyond a complex sense-making process in moments of vulnerability. As also suggested by Foucault, the constant exercise of self-writing drives a whole active process of self-transformation and self-improvement. Ken’s writing, for example, functions as a drive toward the self-training formulated by Rainer Maria Rilke in the imperative “You must change your life” (cited in Sloterdijk, 2014). Therefore, this look to the future implies for the diarist a desire for qualitative transformation, a desire to change his life and to take control of his own development. In Vygotskian terminology, we could say that the practice of diary writing provides Ken with a zone of proximal development. In this zone, Ken has the opportunity to establish not only a dialogical space, but also a space of probationary action, like a laboratory of experience, in which he can actively rehearse commitments on his future behavior and develop conjectures, sketches, or drafts about himself and his life trajectory.

However, we cannot know exactly the extent to which the practice of diary writing is directly responsible for the personal transformations it helps to set in motion. We suggest that Jeanne’s regular and constant writing has perhaps played an important and even decisive role in her ability to distance herself from the past and reconceptualize her suicide attempt and experience in the psychiatric ward. However, her change of perspective may well be due to the passage of time or to other unknown factors of a very different nature. Nor can we know the extent to which the diary has contributed decisively to Ernest’s retirement experience or to the success or failure of Ken’s commitments.

In any case, our exploratory study may suggest that the practice of diary writing is more important for the process – the continuously renewed and updated future horizon that it opens for the diarist – than for the results derived from it. Diary writing is a work-in-progress on ourselves, a mediational and cultural technology for understanding, articulating, orienting, and transforming our own experience. In short, it is a form of sense-making and self-transformation in times of vulnerability.

In conclusion, the proposal we have developed in this article to approach diaries as technologies underlines the need for psychology to address the question of human agency and how humans make sense and transform the world they live in. An agency that we can acquire, as Bruner (1996, pp. 92–93) reminded us, through dialogic and discursive processes such as those we have seen in our work. The person is making a diary which is, in turn, a way of orienting, making, and transforming herself beyond the pages of the diary. Therefore, the study of the technologies that some people use to live life and at the same time try to understand it, to act and observe themselves, requires trying to understand the living and evolutionarily open character of these technologies and how their different uses drive human development. Our study has provided an analysis of three uses of diary writing in moments of vulnerability. We would like this to serve as an inspiration for future research of other uses of the diary beyond those analyzed here, thus deepening the “irreducible tension” pointed out by Wertsch (1998, p. 180) between cultural tools and active agents.
